# Intrinsic mechanisms of right ventricular autoregulation

**DOI:** 10.1038/s41598-024-59787-w

**Published:** 2024-04-23

**Authors:** Jan-Pit Meinert-Krause, Mare Mechelinck, Marc Hein, Moriz A. Habigt

**Affiliations:** Faculty of Medicine, Anaesthesiology Clinic, University Hospital RWTH Aachen, RWTH Aachen University, Pauwelsstr. 30, 52074 Aachen, Germany

**Keywords:** Cardiovascular biology, Cardiovascular biology

## Abstract

To elucidate the adaptation of the right ventricle to acute and intermittently sustained afterload elevation, targeted preload reductions and afterload increases were implemented in a porcine model involving 12 pigs. Preload reduction was achieved via balloon occlusion of the inferior vena cava before, immediately and 5 min after acute afterload elevation induced by pulmonary artery occlusion or thromboxane A2 analog (U46619) infusion. Ventricular response was monitored by registration of pressure–volume (PV) loops using a conductance catheter. The end-systolic pressure–volume relationship (ESPVR) during pure preload reduction was adequately described by linear regression (mean and SEM slope of ESPVR (Ees) 0.414 ± 0.064 mmHg/ml), reflecting the classical Frank-Starling mechanism (FSM). The ESPVR during acute afterload elevation exhibited a biphasic trajectory with significantly distinct slopes (mean and SEM Ees bilin1: 1.256 ± 0.066 mmHg ml; Ees bilin2: 0.733 ± 0.063 mmHg ml, p < 0.001). The higher slope during the first phase in the absence of ventricular dilation could be explained by a reduced amount of shortening deactivation (SDA). The changes in PV-loops during the second phase were similar to those observed with a preload intervention. The persistent increase in afterload resulted in an increase in the slopes of ESPVR and preload recruitable stroke work (PRSW) with a slight decrease in filling state, indicating a relevant Anrep effect. This effect became more pronounced after 5 min or TXA infusion. This study demonstrates, for the first time, the relevance of intrinsic mechanisms of cardiac autoregulation in the right ventricle during the adaptation to load. The SDA, FSM, and Anrep effect could be differentiated and occurred successively, potentially with some overlap. Notably, the Anrep effect serves to prevent ventricular dilation.

## Introduction

Several mechanisms play a role in the adaptation of ventricular work to volume or pressure load. These include not only control by the autonomic nervous system^1–3^, but also intrinsic autoregulatory mechanisms^4–6^. The right ventricle (RV) has been rarely investigated in terms of this matter, in contrast to the left ventricle (LV), which has been extensively covered in the literature.

Previous studies on this topic show that although there are parallels in left and right ventricular adaptation to volume and pressure changes, there are also relevant differences^7,8^. The fast response to load or Frank-Starling mechanism (FSM) appears to play a role in the RV^9–11^, where an increase in ventricular filling leads to an increase in the work done between consecutive beats. Contractility (the relation of work to load) remains constant. But the effect does not appear to play as important a role in long-term adaptation to increased afterload as in the LV^11,12^. Correspondingly, several studies have shown that the RV does not dilate significantly during prolonged afterload increases (> 15 min)^9,12–15^.

This lack of ventricular dilation cannot be explained with certainty solely by a low FSM, but perhaps rather through the slow response to load or the Anrep effect. In the LV, the Anrep effect leads to increased contractility of the heart during phases of persistent dilation, mainly triggered by an increase in afterload. This results in ventricular unloading to the initially load level^5^.

In a previous study, we demonstrated that in the LV, in addition to the FSM and the Anrep effect, a very short-term effect plays an important role in the adaptation to acutely increased afterload^16^. The effect amplifies ventricular force when there's an acute increase in afterload, consequently leading to a diminished ejection velocity necessary to achieve an equivalent level of muscle shortening. We refer to this effect as “reduced amount of shortening deactivation” (SDA)^6,17^. It is unknown whether this effect is also present in the RV. Previous studies investigating adaptation to increased afterload in the RV^9,12–15^ have focused on long-term effects due to the study design. In contrast, we also analyzed the immediate effects of load interventions on ventricular function to describe the specificities of FSM, Anrep, and SDA in the RV. To this end, we performed targeted changes in RV preload and afterload in a porcine animal model and examined the short- (~ 10 s) and long-term (5 min) effects on the ventricle and hemodynamics.

## Materials and methods

### Animals and maintenance of anesthesia

The procedures described below were analogous to the approach in our previous work on the LV^16^. Twelve female German Landrace pigs (sus scrofa domesticus) weighing 47.52 kg ± 5.01 kg were subjected to intramuscular premedication. Premedication consisted of the administration of 4 mg/kg body weight (BW) azaperone (Elanco Tiergesundheit AG, Basel, Switzerland). Subsequently, the pigs were anesthetized using 3 mg/kg BW of propofol (Hexal AG, Holzkirchen, Germany) for oral intubation. Anesthesia was maintained by insufflation of 0.9–1.2 vol% isoflurane and continuous administration of 6–8 µg/kg BW/h fentanyl. Normoventilation was ensured using the Cato ventilator (Drägerwerk AG, Lübeck). The animals were initially ventilated with a tidal volume of 6–8 ml/kg body weight, a respiratory rate of 12/min, and a PEEP of 5 mmHg. The initial oxygen concentration in the inspired air was set at 40%. Regular blood gas analysis checks were performed, and adjustments to the ventilation parameters were made to maintain the values obtained in the blood gas analysis within a physiological range. A balanced crystalloid solution (Sterofundin Iso Braun^®^, Melsungen, Germany) was infused at a rate of 6–10 ml/kg BW/h to ensure a stable fluid balance. An additional dose of balanced colloid solution (Gelafundin Iso Braun^®^) was infused in case of blood loss or volume deficiency, which were characterized by low cardiac output and hypotension. Throughout the procedure, the body temperature was carefully maintained at 38 °C using an airflow warming blanket.

### Surgical instrumentation

**Figure 1:**
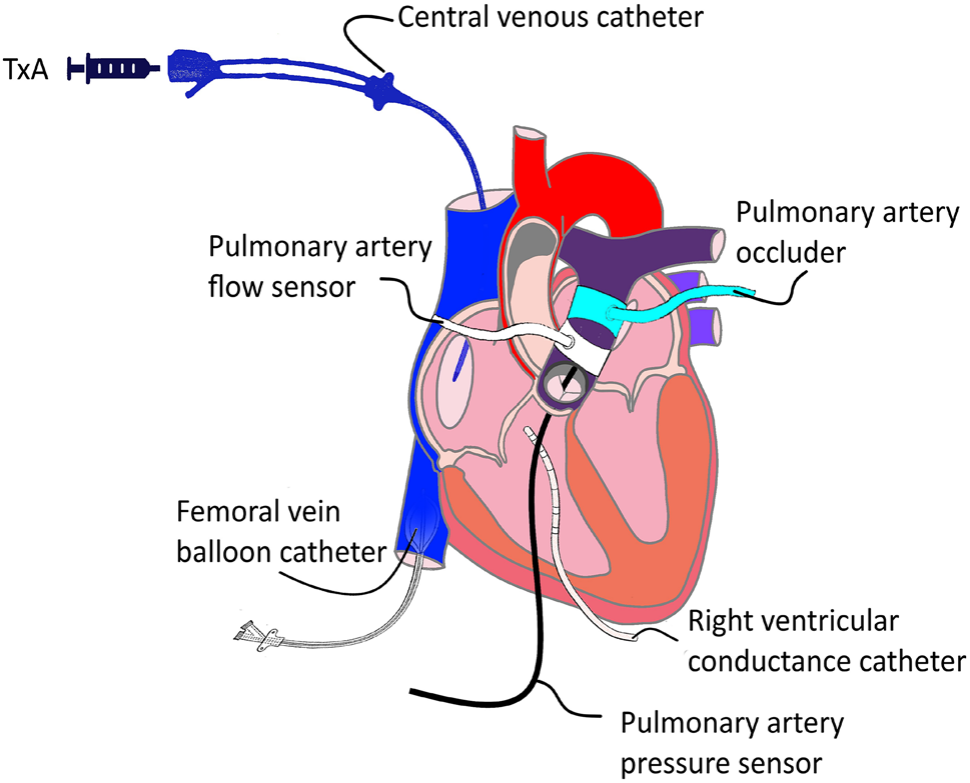
Schematic instrumentation of the right ventricle with ventricular pressure and volume measurement (conductance catheter), pressure and flow measurement in the pulmonary artery, balloon catheter for preload reduction, pulmonary artery occluder for afterload increase and central venous catheter for application of the Thromboxane A2 analog U46619 (TxA).

A central venous catheter was inserted into the right internal jugular vein to infuse the fluids. After a median thoracotomy, the aorta and pulmonary artery were separated. Then a perivascular ultrasound transit-time flow probe (MA 20 PAX; Transonic Systems Europa, Maastricht, the Netherlands) was positioned around the pulmonary artery (see Fig. 1) and connected to a flow meter (T402-PV, Transonic Systems Europa). After pericardiotomy, a solid-state pressure sensor (CA-61000-PL, CD Leycom, Zoetemeer, the Netherlands) was inserted into the RV through a small puncture site. This sensor was advanced through the pulmonary valve until its tip lay 3–4 cm distal to the respective valve in the pulmonary artery. The sensor was then connected to a pressure interface (Sentron, CD Leycom). For additional measurements, a multisegment dual-field 7F conductance catheter (SPR-570-7; Millar Instruments, Houston, TX, USA) was employed, which was inserted through another puncture site in the right ventricular apex. The catheter tip was placed in the right ventricular outflow tract. Echocardiographic imaging was conducted to verify the precise positioning of the catheter longitudinally in the RV. Subsequently, the catheter was connected to a signal processor (Sigma-5 DF, CD Leycom) and a pressure interface (PCU-2000; Millar Instruments) to acquire the instantaneous RV volume and pressure. Then, a 10 French (10F) sheath was inserted into the right femoral vein and a 8 *F* balloon catheter (62080822F, Edwards Lifesciences, Irvine, CA, USA) was positioned within the inferior vena cava. Last, an inflatable perivascular occluder was placed distal to the flow sensor around the pulmonary artery (vascular occluder 20 mm, Invivometric, Healdsburg, California).

Once the instrumentation was completed, no further manipulation was performed on the animals for 30 min under continued isoflurane and fentanyl narcosis to achieve stable blood pressure, cardiac output (CO), and normothermia.

### Interventions

**Figure 2:**
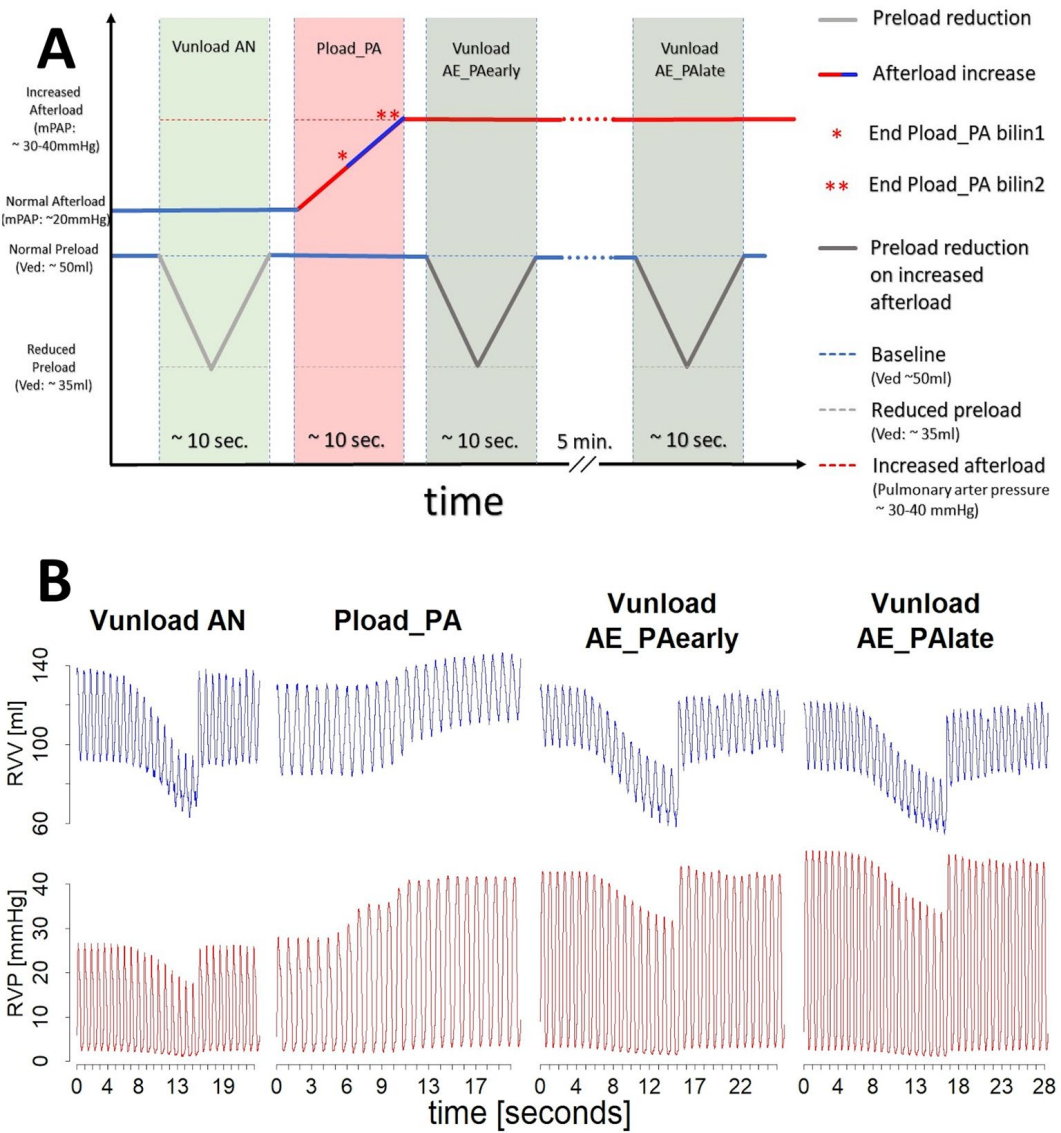
Schematic illustration (**A**) and exemplary animal data (**B**) of the load interventions: preload reduction on a normal afterload level (Vunload AN) followed by an acute afterload increase by pulmonary artery occlusion (Pload_PA), a preload reduction on the elevated afterload level directly after the afterload increase (Vunload AE_PAearly) and finally a preload reduction on the elevated afterload after 5 min. of afterload elevation (Vunload AE_PAlate).

If this manuscript refers to pre- or afterload changes, this always applies to the RV unless otherwise stated. Different types of interventions were applied: preload decrease by reduction of venous return (Vunload) on normal afterload (AN), afterload increase by increase of pulmonary artery pressure (PAP) (Pload_PA), reduction in preload at increased afterload (afterload elevated: AE) immediately after the initial increase in afterload (Vunload AE_PAearly) and 5 min after the increase in afterload (Vunload AE_PAlate). A schematic illustration of the load interventions and exemplary animal data is given in Fig. 2. In addition, RV afterload was increased by continuous infusion of the thromboxane A2 analog U46619 (AE_TxA; Cayman Chemical, Ann Arbor, MI, USA) to demonstrate the differences between different methods to increase right ventricular afterload. Preload reduction was also performed at this increased afterload level (Vunload AE_TxA) after reaching a steady state.

Preload reductions (Vunload) were achieved by brief (~ 10 s) inflation of a balloon catheter in the inferior vena cava. As mentioned, preload reductions were performed at normal and elevated afterload levels immediately (Vunload AE_PAearly) and 5 min after the initial afterload increase (Vunload AE_PAlate).

**Figure 3:**
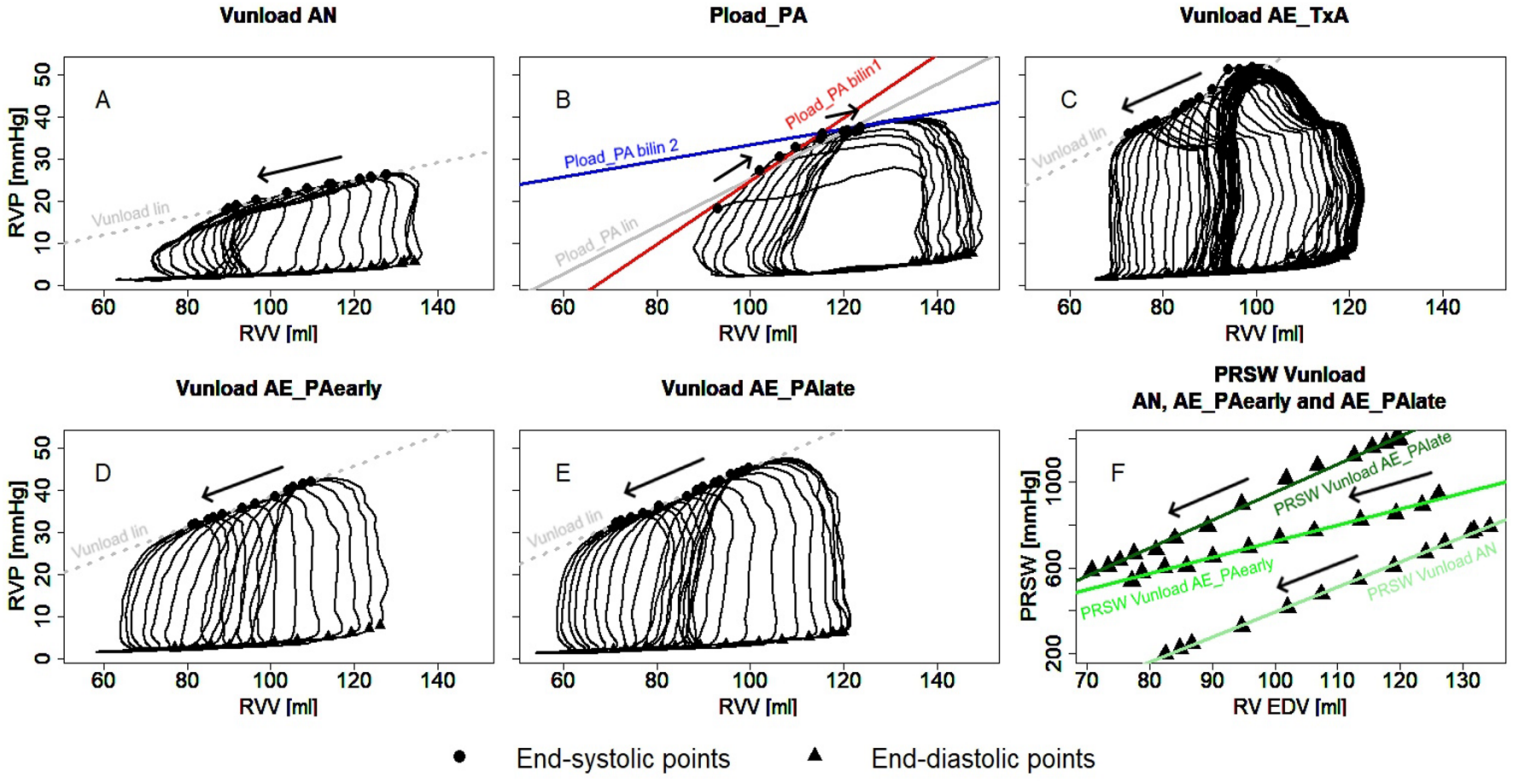
Examples of pressure–volume loops during acute preload reduction on normal afterload (Vunload AN, **A**), acute afterload increase (Pload_PA, **B**), preload reduction while afterload increase by thromboxane A2 analog infusion (Vunload AE_TxA, **C**) and preload reduction directly (Vunload AE_PAearly, **D**) and 5 min after preload increase (Vunload AE_PAlate, **E**). A linear end-systolic pressure‒volume relationship (ESPVR) is shown for all interventions (lin) and a bilinear ESPVR for the afterload intervention (Pload_PA bilin1/2). Moreover, the preload recruitable stroke work (PRSW) for the Vunload AN, AE_PAearly and AE_PAlate interventions is displayed (**F**). The arrows illustrate the direction of movement of the loops during the respective interventions (*RVV* right ventricular volume, *RVP* right ventricular pressure).

Elevated afterload was achieved by partial inflation of an occluder around the pulmonary artery to achieve a systolic PAP of 30–40 mmHg for ~ 10 s. The position and filling volume of the balloon catheter were maintained for all 3–6 repetitions and during all interventions (Vunload and Pload_PA) in all animals. A subsequent intervention was only performed once the baseline values from the period prior to the previous intervention were fully restored. Exemplary pressure–volume loops (PV loops) for all interventions are shown in Fig. 3.

### Data acquisition and calculations

Continuous signals were acquired at a sampling rate of 1 kHz using a data acquisition device (Powerlab, AD Instruments, Dunedin, New Zealand) and LabChart software (AD Instruments, Dunedin, New Zealand). LabChart's built-in cycle detection algorithm, which relies on the R-wave of the ECG signal to define end-diastole, was used to identify individual beats. Subsequently, signals of approximately 20 s duration centered around the interventions were exported for further processing using R in RStudio (version 4.2.2)^18,19^ and several additional packages (“*tidyverse*”, “*pracma*”, “*nlsr*”)^20–22^. Extrasystoles were manually excluded. To correct the volume signal, the parallel conductance (calculated from the recordings during venous hypertonic saline injections) and the slope factor α (which represents the ratio of the stroke volume from the conductance signal and the aortic flow probe) were applied as previously described^13,23,24^. An algorithm specifically developed to determine end-systole in the often triangular right ventricular pressure‒volume loops was selected^25,26^. This algorithm calculates the theoretical isovolumic pressure of each beat and combines it with the actual maximum ventricular volume. End-systole was determined by drawing a tangent through the upper left corner of the PV loop at this point^4,27^.

From changes in end-systolic volume and pressure during load intervention, the end-systolic pressure‒volume relationship (ESPVR) was calculated to describe contractility. Two types of regression were used to describe the ESPVR. First, a single linear regression was performed through the ESPVR points of the entire intervention to calculate the slope (Ees lin) and x-intercept (V0 lin), as well as the Pearson correlation coefficient (r) as a marker of goodness of fit. Second, a ‘bilinear ESPVR’ was determined by selecting the combination of heartbeats for which two linear regressions through the end-systolic points with the highest geometric mean of the two r values of both regressions were present (bilin1 and bilin2). The intersection of the two regression lines defined the end of the first segment (end bilin1). Figure 3 illustrates exemplary pre- and afterload interventions with the three different associated regression lines for the ESPVR. To describe the effect of afterload level on contractility, the slope (Mw) of the preload recruitable stroke work was calculated from linear regression analysis of end-diastolic volume and stroke work obtained from preload interventions.

In addition, selected parameters were determined for the specific beat at the following time points: just before the start of bilin1 (baseline), at the end of bilin1 (end bilin1), and at the end of bilin2 (end bilin2). The parameters selected were end-systolic pressure (Pes), maximum pressure rise per time unit (dP/dtMax), stroke work (SW), end-diastolic and end-systolic volumes (Ved and Ves), and stroke volume (SV). Additionally, the subsequent parameters were computed as follows: the passive (stiffness) components of ventricular relaxation were displayed by the exponential regression of the end-diastolic pressure volume points (end-diastolic pressure volume relationship: EDPVR), which was characterized by the EDPVR indices P0, V0 and λ (${\text{P}}\_\mathrm{ED }=\mathrm{ P}0\times ({\text{exp}}(\uplambda ({\text{V}}\_{\text{ED}}-{\text{V}}0)))-1$). We iteratively calculated the single indices of the equation. The compliant characteristics (C) of the Windkessel vessels were calculated by dividing the SV by the difference in systolic and diastolic pressure (pulse pressure [PP]) as previously described^28^. Fourier series expressions for pressure and flow signals of 20 s duration were used to calculate systemic vascular resistance and impedance. The impedance modulus at each frequency was calculated as the ratio between pressure and flow moduli (amplitudes). The total resistance (R) and characteristic impedance (Zc) were derived from moduli at zero frequency and the average of moduli between 2 and 15 Hz^29^.

### Statistics

We used SPSS 24.0 software (IBM Corporation, Armonk, USA) to perform statistical tests and paint.net (Rick Brewster, dotPDN, L.L.C., San Francisco, USA), R and RStudio^18,19^, and Prism (PRISM 8.1, GraphPad Software, San Diego, USA) to create graphs and visualize data. We present the mean and standard error of the mean (SEM) of the intervention outcomes.

ESPVRs were excluded if r < 0.8, Ees bilin1 − Ees bilin2 < 0, or Ees < 0 were present.

The effect of intervention type (Vunload, Pload_PA) on the Ees and r of different ESPVRs and other hemodynamic parameters was analyzed by a generalized linear mixed model to be able to consider repeated measurements. Additional pairwise estimations of contrasts were defined. A value of p < 0.05 was considered statistically significant.

### Ethics approval and consent to participate

All applied procedures are compliant with the Guide for the Care and Use of Laboratory Animals^39^ and the ARRIVE Guidelines^40^ and have been reviewed and approved by the local animal care committee and the governmental animal care office (No. 84-02.04.2013. A476; Landesamt für Natur-, Umwelt- und Verbraucherschutz Nordrhein-Westfalen, Recklinghausen, Germany).

## Results

**Figure 4:**
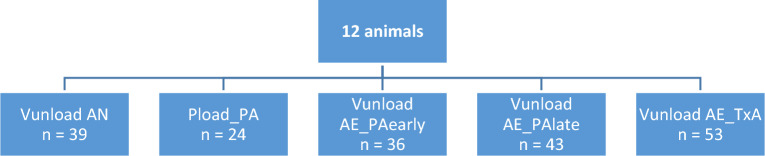
Number of interventions evaluated from a total of 12 animals.

Interventions performed on all animals could be used for analysis. In detail, 39 of 48 Vunload interventions at normal afterload, 36 of 39 at Vunload AE_PAearly, and 43 of 52 at late afterload intervention could be included. From Pload_PA interventions, 24 of the original 59 interventions could be further evaluated. After TxA administration, 53 of the original 59 interventions met the conditions (see Fig. 4). Exclusion criteria included poor signal quality due to rhythm disturbances, artifacts caused by the conductance catheter touching the ventricular wall, or issues during interventions, such as excessive ventricular volume unloading.

### Linearity of the end-systolic pressure‒volume relationship

**Figure 5:**
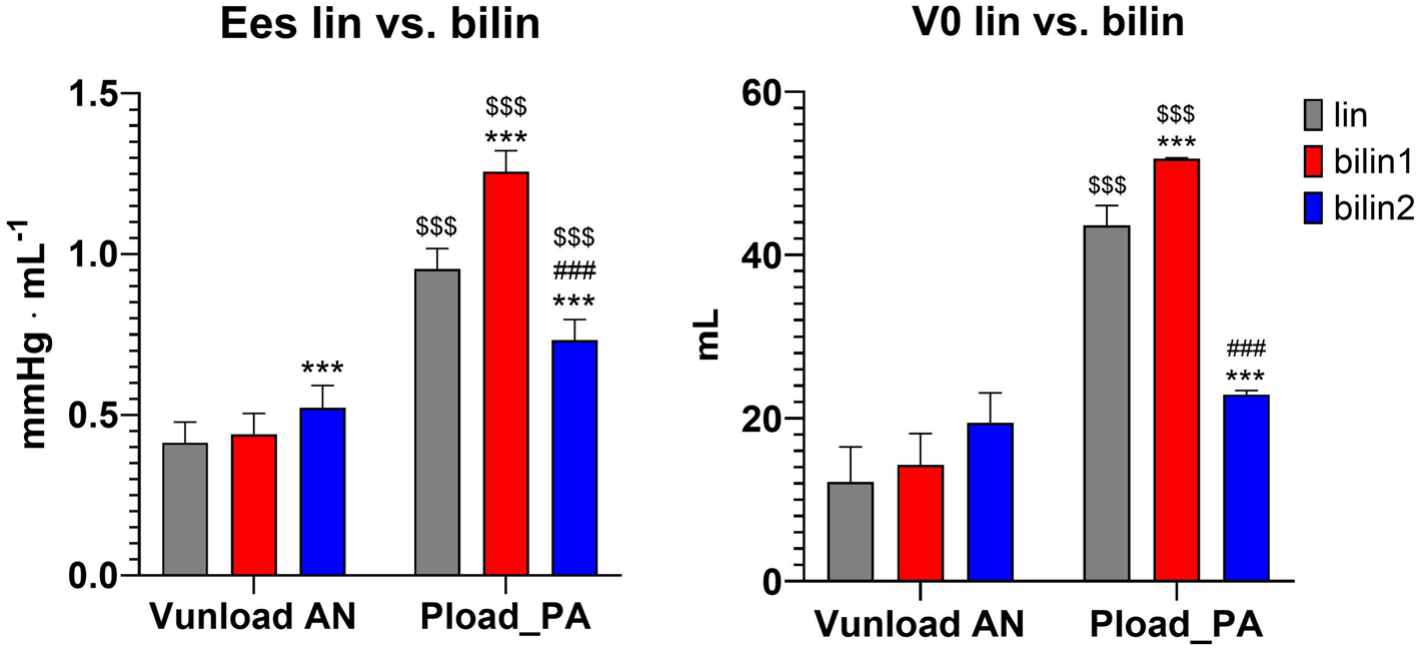
Slopes (Ees) and x-intercepts (V0) of end-systolic pressure‒volume relationship (ESPVR) during change of preload (Vunload) and afterload (Pload_PA). Each shown as single linear (Ees lin), as well as bilinear regression. For the bilinear regression, the slope of the initial phase (Ees bilin1) and the slope of the consecutive 2nd phase (Ees bilin2) are shown. The numerical values of these results are presented as supplementary materials in Supplementary Table S1 (Mean values ± SEMs: ***p < 0.001 vs. lin; ^###^p < 0.001 vs. bilin1; ^$$$^p < 0.001 vs. V_unload_IVC).

ESPVR was nonlinear during Pload_PA but linear during Vunload. During Pload_PA, the mean Ees bilin1 and Ees bilin2 values differed significantly from the Ees lin values (see Fig. 5 and Supplementary Table S1 of the supplementary materials). The initial Ees bilin1 value was significantly higher than the following Ees bilin2 value.

There was no significant difference between Ees bilin1 and Ees bilin2 values during Vunload. There was only a slight but significant increase in Ees bilin2 values compared to Ees lin during Vunload. In general, significantly higher values of Ees lin, Ees bilin1 and Ees bilin2 were observed during Pload_PA than during Vunload. This was accompanied by a significantly higher V0 during Pload_PA than during Vunload. The V0 of Pload_PA bilin1 was significantly higher than that of Pload_PA bilin2. Thus, afterload interventions led to a higher slope and rightward shift of the ESPVR, especially during the initial phase.

### Fast response to load/shortening deactivation

**Figure 6:**
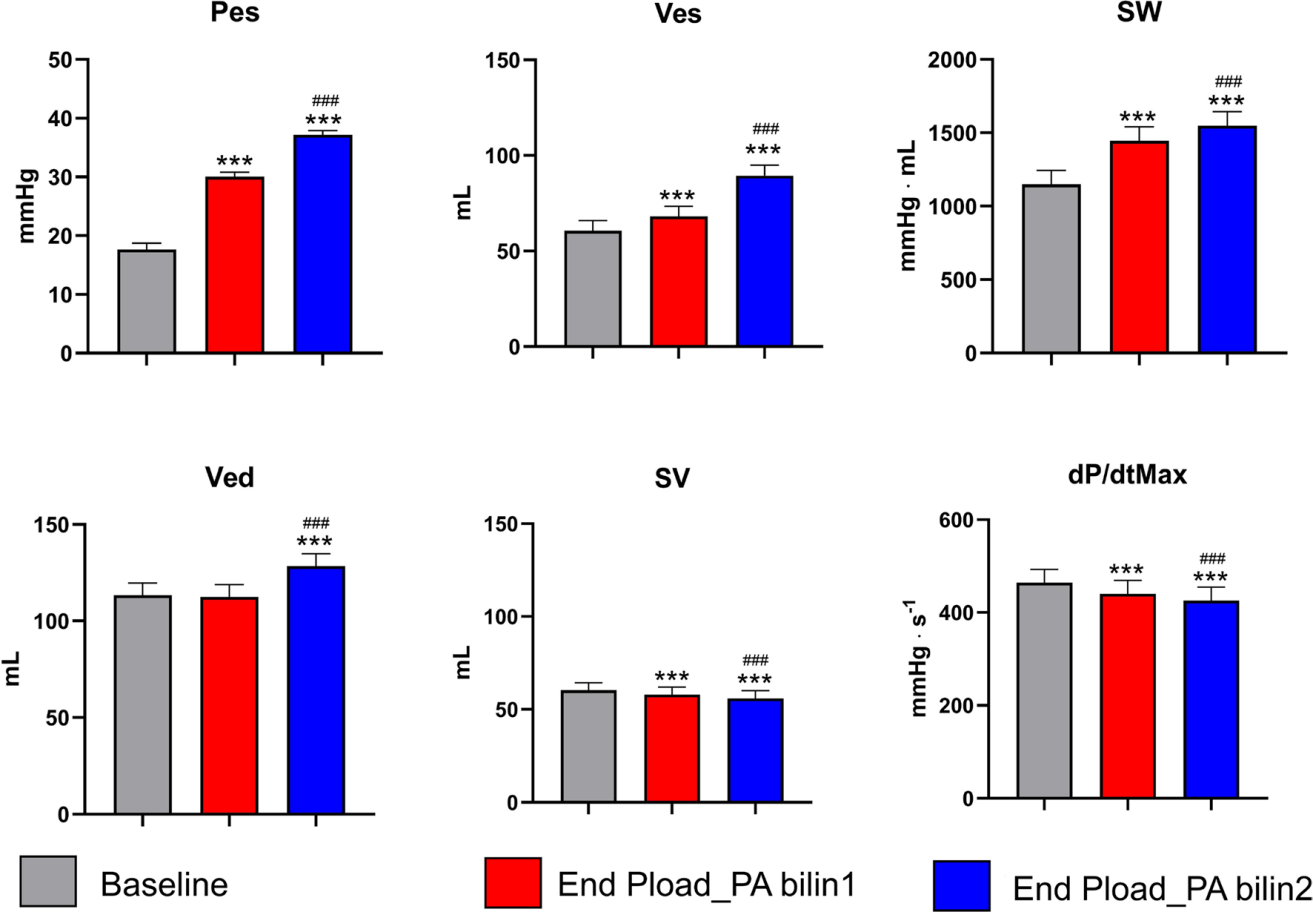
Effect of afterload intervention on selected ventricular parameters (end-systolic pressure [Pes] and volume [Ves], stroke work [SW], end-diastolic volume [Ved], stroke volume [SV] and maximal pressure rise per time unit [dP/dtMax]) at different time points: Values at the end of first (end Pload_PA bilin1) and second (end Pload_PA bilin2) end-systolic pressure volume relationship (ESPVR) were compared with values before the intervention (Baseline). The numerical values of these results are presented as supplementary materials in Supplementary Table S2 (Mean values ± SEMs: ^***^p < 0.001 vs. baseline ^###^p < 0.001 vs. end P_load_PA bilin1).

Pes increased significantly at end Pload_PA bilin1 compared to baseline, followed by a further increase toward end Pload_PA bilin2. Ved only increased significantly at end Pload_PA bilin2 compared to baseline and end Pload_PA bilin1. Ves continuously increased significantly during both phases of Pload_PA compared to baseline. In contrast, SV continuously decreased significantly during the afterload intervention. While SW, analogous to Pes and Ves, increased significantly in both phases of Pload_PA, dP/dtMax decreased significantly (see Fig. 6 and Supplementary Table S2 of the supplementary materials).

### Slow response to load

**Figure 7:**
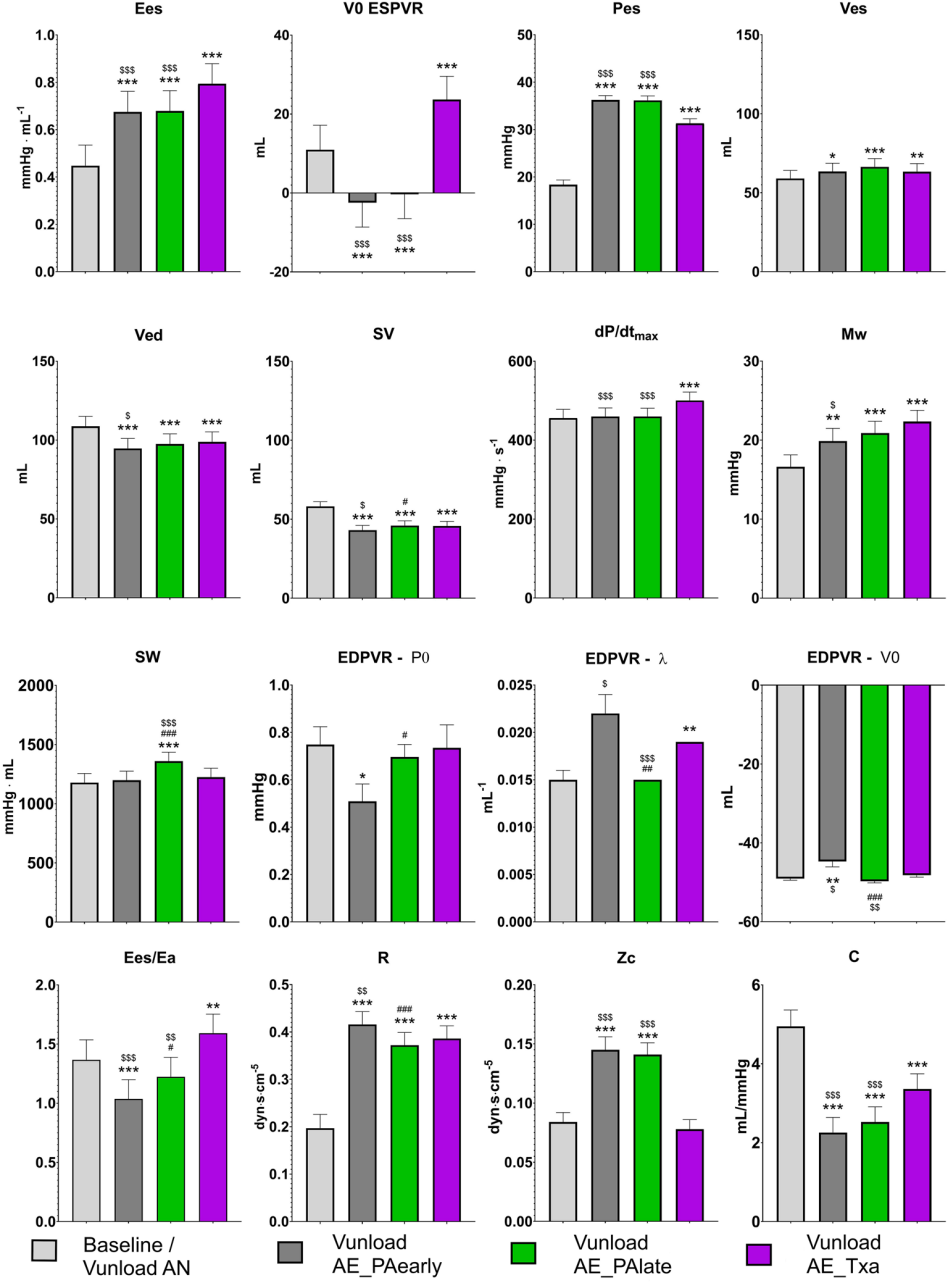
Effect of increased afterload over time (AE_PAearly and -late) and with respect to type of intervention (pulmonary artery occlusion (AE_PA) vs. Thromboxane analog infusion (AE_TxA) on various parameters compared to the normal afterload state (baseline or preload reduction (Vunload AN)). The parameters shown are the slope [Ees] and volume-axis intercept [V0 ESPVR] of the end-systolic pressure volume relationship obtained during preload interventions and the following selected hemodynamic parameters obtained during baseline: end-diastolic volume [Ved], end-systolic pressure [Pes] and volume [Ves], stroke volume [SV], stroke work [SW], maximum pressure rise per time unit [dP/dtMax] and slope of the preload recruitable stroke work [Mw]. Parameters for identifying the parabolic end-diastolic pressure–volume relationship (EDPVR [P0, λ, V0]), the arterioventricular coupling [Ees/Ea] and the pulmonary vascular bed [R, Zc, C] are presented. The numerical values of these results are presented as supplementary materials in Supplementary Table S3 (Mean values ± SEMs: *p < 0.05, **p < 0.01, ***p < 0.001 vs. baseline; ^#^p < 0.05, ^###^p < 0.001 vs. AE_PAearly; ^$^p < 0.05, ^$$$^p < 0.001 vs. AE_TxA).

The significantly increased mean Ees and Pes values that were achieved during the Pload_PA intervention were maintained during the Vunload AE_PAearly/late interventions compared to baseline (Fig. 7 and Supplementary Table S3 of the supplementary materials). However, 5 min after reaching the increased afterload level, no further significant changes in either parameter could be measured on average (Ees/Pes high late compared to Ees/Pes high early). The increase in afterload due to TxA infusion also resulted in a significant increase in Ees and Pes, with on average significantly higher Ees and significantly lower Pes values, compared to the values with increased afterload due to aortic occlusion (Vunload AE_PAearly/late). In contrast, V0 exhibited significant differences in the response to the increase in afterload due to aortic occlusion (Vunload AE_PAearly/late) compared to the increase in afterload due to TxA infusion. While aortic occlusion led to a leftward shift of the ESPVR curve, TxA infusion caused a rightward shift.

Ventricular contractility parameters, including Mw and dP/dtMax, significantly increased during the Vunload AE_TxA intervention in comparison to the baseline and the Vunload AE_PAearly intervention. However, during the Vunload AE_PAearly/late interventions only a significant increase in Mw could be observed compared to baseline. In contrast, SW was significantly elevated only during the Vunload AE_PAlate intervention compared to all other long-term interventions.

There was a significant reduction in both Ved and SV compared to baseline for all variants of afterload increase, with the lowest values at Vunload AE_PAearly, despite a significant increase in Ved observed at end Pload_PA bilin2 (Fig. 6 and Supplementary Table S2 of the supplementary materials). Accordingly, Ves was increased in all interventions compared to baseline.

Generally, there was an increase in the curvature of the parabolic EDPVR curve during all afterload interventions (increase in λ during Vunload AE_PAearly and increase in P0 in Vunload AE_PAlate and AE_TxA) compared to baseline (Fig. 7). The Ees/Ea ratio as a marker of the arterioventricular coupling was shifted towards Ea compared to the baseline during pulmonary artery occlusion experiments (AE_PAearly/-late), whereas the ratio shifted towards Ees during TXA infusion (AE_TxA). This was also reflected in the values characterizing the vascular bed of the pulmonary circulation. There was a significant increase in total resistance (R) during both afterload-increasing experiments (AE_PAearly/-late and AE_TxA), with specific impedance (Zc) increasing significantly only in the AE_PAearly/-late interventions. Compliance (C) significantly decreased in all afterload-increasing experiments, but significantly less during TxA infusion (AE_TxA) compared to pulmonary artery occlusion (AE_PAearly/-late) (Fig. 7).

## Discussion

With the data shown, we could demonstrate the existence of SDA, FSM and an Anrep effect in the RV.

**Figure 8:**
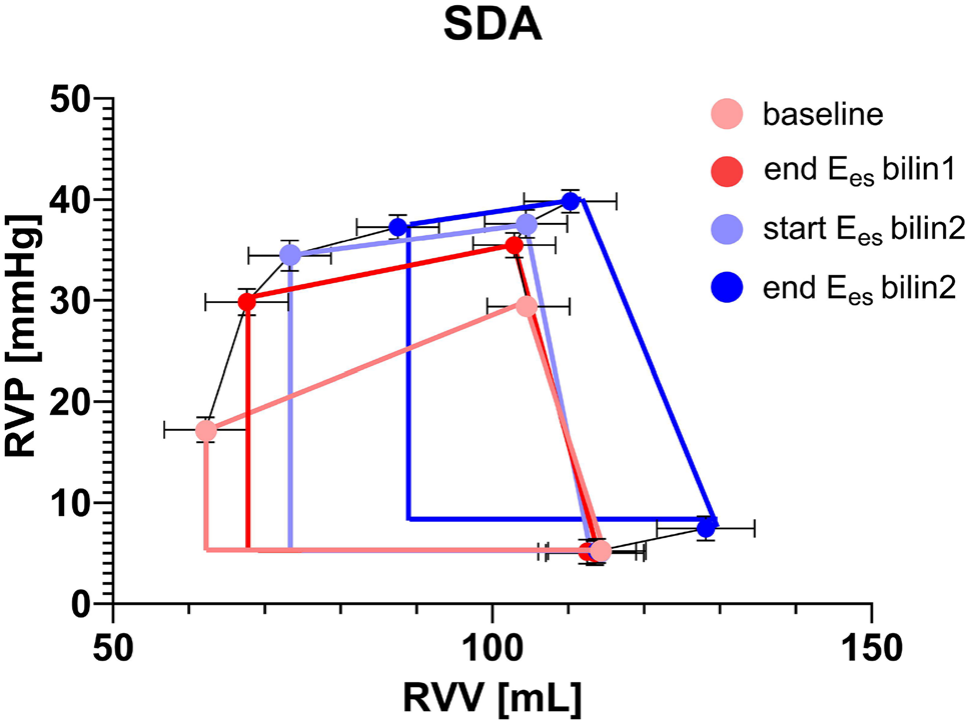
Plot of averaged pressure/volume points (V/Ped, V/Pea = pressure/volume point at the end of isovolumetric contraction & V/Pes) at baseline (light red), last value before the intersection of Ees1 and Ees2 (end Pload_PA 1, dark red) and the first value after the intersection of Ees1 and Ees2 (light blue) and at the end of afterload intervention (end Pload_PA 2, dark blue). *RVP* right ventricular pressure, *RVV* right ventricular volume.

The slope of the RV ESPVR during acute preload interventions can be described as linear, whereas during acute afterload interventions, two phases could be detected (Figs. 3, 5). These results are consistent with the previously published observations for the LV^16^. At the end of Pload_PA bilin1, increased Pes and SW values and decreased SV values were measured, without a corresponding increase in Ved at this time (Figs. 6, 8). This phase of ventricular adaptation to an acute increase in afterload cannot be explained by the FSM. Thus, another mechanism must be at work. According to known mechanisms of cardiac autoregulation, the behavior could be explained by the Gregg phenomenon, the Gardenhose effect or a SDA. Since the right ventricular afterload increase is likely to lead to decreased rather than increased coronary perfusion and increased coronary perfusion has less effect on right ventricular performance than is the case in the left ventricle^30,31^, we consider the influence of both the Gregg phenomenon and the Gardenhose effect in the RV to be insufficient to explain the above results. In view of a stable heart rate during the preload and pulmonary artery occlusion interventions, a sympathetic or parasympathetic activation seems unlikely as well (AN: 87.8 ± 11 bpm, AE_PAearly: 88.4 ± 10.9 bpm, AE_Palate: 88.81 ± 12.88 bpm). Accordingly, we consider a lower amount of SDA to be the causative factor. In the second phase (end Pload_PA bilin2), ventricular dilatation occurs (Figs. 6, 8), which activates the FSM, as evidenced by a sustained increase in Pes and SW. The existence of FSM in the RV is well established in the literature^9,12,32,33^. Thus, the first linear phase of the ESPVR could be assigned to SDA and the directly following phase to the FSM.

**Figure 9:**
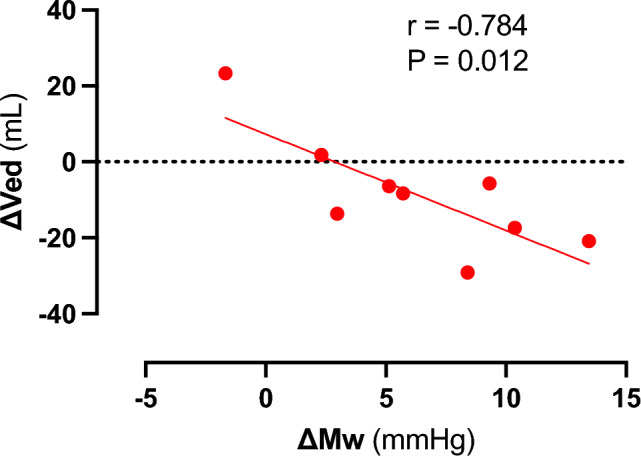
Correlation of changes in contractility as displayed by slope (Mw) of the preload recruitable stroke work (PRSW) and end-diastolic volume (Ved) from baseline to 5 min of high afterload (AE_Palate) in individual animals.

In the case of sustained afterload increase (Vunload AE_PAlate), we observed a classical Anrep effect with increasing right ventricular pressure and simultaneously decreasing right ventricular volume in individual animals (see Figs. 2B, 3D,E). However, we were unable to statistically demonstrate this effect significantly across all animals on average. Nevertheless, we are convinced that our data prove the existence of the Anrep effect in the RV: In the absence of ventricular dilatation, there was an increase in contractility (increase of SW and Mw, Vunload AE_PAlate Fig. 7 and Supplementary Table S3). A simple explanation would be that the occlusion catheters, used to induce the increase in afterload, were unable to maintain a constant level of occlusion. As a result, in some interventions, there was a slight drop in pressure or a less pronounced increase within 5 min. Another possible explanation would be an early onset of the Anrep effect, as an increase in the slope of the PRSW (Mw) was already observed in the Vunload AE_PAearly phase. This early increase did not occur in our previous study on the LV^16^. A further hint is the already decreased Ved in the Vunload AE_PAearly phase. In summary, the following effects might confirm an early Anrep effect: an increase in contractility (Ees and Mw) shortly after the initial afterload increase (Vunload AE_PAearly), which should be stable in FSM, and a lack of ventricular dilation in the Vunload AE_PAearly phase. Together with that, in some animals, a more distinct Anrep effect could be demonstrated (Figs. 2B, 3D,E, 9), suggesting significant interindividual variability. As depicted in Fig. 9, the increase in contractility (change in Mw) from baseline to 5 min of high afterload (AE_PAlate) correlated with a decrease in Ved. The increase in contractility due to the Anrep effect resulted in ventricular unloading.

Nevertheless, the onset of Anrep has been discussed in the literature to occur only after 10–15 min^5,34^, but in previous studies of our group the effect in the LV was observed at a much earlier time point, which prompted us to select an intervention period of five min. It is therefore also possible that the chosen intervention time was too brief to fully observe the expression of the Anrep effect in the RV, but this explanation seems less likely to us, as no initial ventricular dilation occurs. The lack of an increase in Pes over time as an expression of a limited Anrep effect with a substantial interindividual variability is one of the major differences to our findings in the LV. Another difference between the RV and LV is that SW increases at Pload_PA bilin1 in the RV with the acute load intervention, which was not observed in the LV^16^. Furthermore, at Pload_PA bilin2, the ESPVR in the RV shifts to the right, while in the LV V0 remains comparable to the baseline level during this phase of the intervention.

The most striking difference between Pload_PA and Vunload AE_TxA to increase afterload, is the greater increase in contractility (greater increase in Ees, greater increase in dP/dtMax, but no difference in SW) with less increase in Pes, in the case of the Vunload AE_TxA intervention. This may be explained by the expected lower arterial compliance (C) and higher impedance (Zc) during the Pload_PA intervention (see Fig. 7). But it cannot be ruled out that additional autocrine/paracrine effects induced by the infusion of the TxA analogue may contribute to the observed effects. Whereas a sympathetic activation seemed unlikely in the pulmonary occlusion experiments due to a stable heart rate, a slight drop in the heart rate could be observed after TxA analoge infusion (AN: 87 ± 11 bpm, AE_TxA: 83 ± 7 bpm). Also, the Gregg phenomenon does not provide a plausible explanation either, as there was no increase in systolic aortic pressure during the interventions (mean ± SEM systolic aortal pressure AN: 73.84 ± 9.13 mmHg, AE_TxA: 70.94 ± 8.14 mmHg). Further, a contractility-enhancing effect of eicosanoids is described in the literature, but appears to be less pronounced in the right heart^35^. Although the experiments were carefully planned and executed, there are several limitations to consider in evaluating the results of our study. For the introduction of the required measurement equipment, we chose an experimental setup with an opened pericardium. This particularly places the RV in a highly unphysiological state and leads to relevant changes in physiological conditions^36,37^. Especially the ventricular interdependence significantly influences the adaption to high afterload. Opening of the pericardium improves filling and function of the left ventricle. Further, the study was conducted on healthy, female pig hearts. Therefore, drawing pathophysiological conclusions is limited or even impossible. Differences may exist compared to male pigs that were not captured by our methodology.

During the afterload increases (Pload_PA), we aimed for a consistent afterload level of 30–40 mmHg pulmonary arterial pressure. The indication of a target range of 10 mmHg already suggests that it was not always possible to achieve the same afterload level. Different afterload levels were not systematically investigated. Moreover, it is possible that it was statistically impossible to demonstrate the Anrep effect because of a pressure drop within the pulmonary arterial occlusion catheter during the prolonged afterload increase (Pload_PA long), which we could not trace due to the lack of continuous pressure measurement within the occlusion catheter.

Additionally, the conditions of End P_load_bilin2 and Pload_PA & Vunload are not directly comparable since there was a time delay of approximately 1 min between the measurement points. During this time, adaptation mechanisms may have occurred (e.g., early onset of the Anrep effect), which could not be comprehended. Concerning the load interventions, a longer intervention time beyond 10 s could have included more beats in the analysis of intervention phases, potentially further enhancing the quality of regressions.

Due to the variable shape of the RV in the PV loop, determining the correct timing of end-systole proved challenging. Some variability in the data could be explained by this circumstance. Even though we assume a very rapid onset of the Anrep effect in the RV, the literature suggests a time frame of 10–15 min. A longer intervention period for the afterload intervention (Vunload AE_PAlate) would have avoided uncertainty in interpreting the results.

The presented results can assist in categorizing clinical findings. For instance, they underscore that a dilated right ventricle must be viewed as a sign of decompensation and not an expression of normal adaptation to increased afterload. The relevant interindividual variability to increased contractility might be crucial for the acute adaptation to high afterload, as described earlier^38^.

## Conclusion

In our experiment, an acute increase in right-ventricular afterload resulted in a biphasic ESPVR. We hypothesize that SDA is the causative factor for the first phase and the FSM for the second phase. The Anrep effect demonstrated significant interindividual variability and probably occurred early, inhibiting ventricular dilation.

### Supplementary Information

Supplementary Table S1.

Supplementary Table S2.

Supplementary Table S3.

## Data Availability

The datasets used and analyzed during the current study are available from the corresponding author on reasonable request.
