# Supplementary material for: Intrinsic mechanisms of right ventricular autoregulation
Source: Sci Rep. 2024 Apr 23;14:9356. doi: 10.1038/s41598-024-59787-w (PMC11039625; doi:10.1038/s41598-024-59787-w)
Supplement: Supplementary file 1 — Supplementary Table S1. [file 41598_2024_59787_MOESM1_ESM.docx]

|  | Vunload AN | | Pload_PA | |
| --- | --- | --- | --- | --- |
|  | *Mean* | *SEM* | *Mean* | *SEM* |
|  |  |  |  |  |
| Ees lin [mmHg/mL] | 0.414 | 0.064 | 0.953 ^$$$^ | 0.064 |
| Ees bilin1 [mmHg/mL] | 0.44 | 0.065 | 1.256 *** ^$$$^ | 0.066 |
| Ees bilin2 [mmHg/mL] | 0.522 *** | 0.07 | 0.733 *** ^### $$$^ | 0.063 |
| V0 lin [mL] | 12.194 | 4.302 | 43.629 ^$$$^ | 2.414 |
| V0 bilin1 [mL] | 14.272 | 3.851 | 51.78 *** ^$$$^ | 0.118 |
| V0 bilin2 [mL] | 19.438 | 3.682 | 22.915 ^### $$$^ | 0.484 |
| *(*** P < 0.001 vs. lin; ### P < 0.001 vs. bilin1; $$$ P < 0.001 vs. Vunload AN)* | | | | |

**Table S1**

**Table S1**: Mean slope (Ees) and x-intercepts (V0) values of end-systolic pressure‒volume relationship (ESPVR) during change of preload (Vunload AN) and afterload (Pload_PA). Values for a single linear (Ees lin), as well as a bilinear regression are presented. For the bilinear regression, the slope of the initial phase (Ees bilin1) and the slope of the consecutive 2nd phase (Ees bilin2) are shown.
