# Supplementary material for: Intrinsic mechanisms of right ventricular autoregulation
Source: Sci Rep. 2024 Apr 23;14:9356. doi: 10.1038/s41598-024-59787-w (PMC11039625; doi:10.1038/s41598-024-59787-w)
Supplement: Supplementary file 2 — Supplementary Table S2. [file 41598_2024_59787_MOESM2_ESM.docx]

|  | Baseline | | End Pload_PA bilin1 | | End Pload_PA bilin2 | |
| --- | --- | --- | --- | --- | --- | --- |
|  | *Mean* | *SEM* | *Mean* | *SEM* | *Mean* | *SEM* |
|  |  |  |  |  |  |  |
| Pes [mmHg] | 17.6 | 1.1 | 30.0 *** | 0.7 | 37.1 *** ^###^ | 0.7 |
| Ves [ml] | 60.5 | 5.3 | 68.1*** | 5.3 | 89.4*** ^###^ | 5.4 |
| Ved [ml] | 113.2 | 6.429 | 112.3 | 6.4 | 128.4 *** ^###^ | 6.4 |
| SW [mmHg/ml] | 1149 | 94 | 1445*** | 94 | 1549 *** ^###^ | 94 |
| SV [ml] | 60.2 | 4.1 | 57.9 *** | 4.1 | 55.9 *** ^###^ | 4.1 |
| dP/dtMax [mmHg/s] | 464.1 | 28.8 | 440.4 *** | 28.8 | 425.8 *** ^###^ | 28.9 |
| *(*** P < 0.001 vs. Baseline ### P< 0.001 vs. end Pload_PA bilin1)* | | | | | | |

**Table S2**

**Table S2**: Effect of afterload intervention on selected ventricular parameters (end-systolic pressure [Pes] and volume [Ves], end-diastolic volume [Ved], stroke work [SW], stroke volume [SV] and maximal pressure rise per time unit [dP/dtMax]) at different time points: Values at the end of first (end Pload_PA bilin1) and second (end Pload_PA bilin2) end-systolic pressure volume relationship (ESPVR) are compared with values before the intervention (Baseline).
