# Supplementary material for: Intrinsic mechanisms of right ventricular autoregulation
Source: Sci Rep. 2024 Apr 23;14:9356. doi: 10.1038/s41598-024-59787-w (PMC11039625; doi:10.1038/s41598-024-59787-w)
Supplement: Supplementary file 3 — Supplementary Table S3. [file 41598_2024_59787_MOESM3_ESM.docx]

|  | Baseline/  Vunload AN | | Vunload AE_PAearly | | Vunload AE_PAlate | | Vunload AE_TxA | |
| --- | --- | --- | --- | --- | --- | --- | --- | --- |
|  | *Mean* | *SEM* | *Mean* | *SEM* | *Mean* | *SEM* | *Mean* | *SEM* |
|  |  |  |  |  |  |  |  |  |
| Ees [mmHg/mL] | 0.44 | 0.08 | 0.67 *** ^$$$^ | 0.08 | 0.67*** ^$$$^ | 0.08 | 0.79 *** | 0.08 |
| V0 ESPVR [mL] | 10.9 | 6.1 | -2.4*** ^$$$^ | 6.1 | -0.3 *** ^$$$^ | 6.1 | 23.6 *** | 5.9 |
| Pes [mmHg] | 18.3 | 0.9 | 36.2 *** ^$$$^ | 0.9 | 36.1*** ^$$$^ | 0.9 | 31.3 *** | 0.9 |
| Ves [mL] | 59.0 | 5.1 | 63.4 * | 5.2 | 66.4 *** | 5.2 | 63.2 ** | 5.1 |
| Ved [mL] | 108.7 | 6.2 | 94.7 *** ^$^ | 6.3 | 97.5 *** | 6.3 | 98.8 *** | 6.3 |
| SV [mL] | 58.1 | 2.9 | 43.1 *** ^$^ | 2.9 | 46.0 *** ^#^ | 2.9 | 45.6 *** | 2.9 |
| dP/dtMax [mmHg/s] | 456.1 | 22.0 | 459.8 ^$$$^ | 21.8 | 459.7 ^$$$^ | 21.2 | 500.6 *** | 21.2 |
| Mw [mmHg] | 16.6 | 1.5 | 19.8 ** ^$^ | 1.6 | 20.8 *** | 1.4 | 22.3 *** | 1.4 |
| SW [mmHg/ml] | 1178 | 76.8 | 1198 | 76.7 | 1359 *** ^###^ ^$$$^ | 75.9 | 1224 | 75.6 |
| EDPVR – P0 [mmHg] | 0.74 | 0.07 | 0.50 * | 0.07 | 0.69 ^#^ | 0.05 | 0.73 | 0.09 |
| EDPVR – λ [ml^-1^] | 0.015 | 0.001 | 0.022 ^$^ | 0.002 | 0.015 ^## $$^ | 0 | 0.019 ** | 0 |
| EDPVR – V0 [ml] | -49.07 | 0.408 | -44.71 ** ^$^ | 1.368 | -49.733 ^### $$^ | 0.406 | -48.218 | 0.474 |
| Ees/Ea | 1.36 | 0.16 | 1.03 *** ^$$$^ | 0.16 | 1.22 ^#^ ^$$^ | 0.16 | 1.59 ** | 0.16 |
| R | 0.197 | 0.029 | 0.416 *** ^$$^ | 0.027 | 0.372 *** ^###^ | 0.027 | 0.386 *** | 0.027 |
| Zc | 0.084 | 0.008 | 0.145 *** ^$$$^ | 0.011 | 0.141 *** ^$$$^ | 0.01 | 0.078 | 0.008 |
| C | 4.94 | 0.41 | 2.25 *** ^$$$^ | 0.38 | 2.52 *** ^$$$^ | 0.38 | 3.36 *** | 0.38 |
| *(* P < 0.05, ** P < 0.01, *** P < 0.001 vs. Baseline; # P < 0.05, ### P < 0.001 vs. Vunload AE_PAearly;  $ P < 0.05, $$$ P < 0.001 vs. Vunload AE_TxA)* | | | | | | | | |

**Table S3**

**Table S3:** Effect of increased afterload over time (AE_PAearly and -late) and with respect to type of intervention (pulmonary artery occlusion (AE_PA) vs. Thromboxane analog infusion (AE_TxA) on various parameters compared to the normal afterload state (baseline or preload reduction (Vunload AN)). The parameters shown are the slope [Ees] and volume-axis intercept [V0 ESPVR] of the end-systolic pressure volume relationship obtained during preload interventions and the following selected hemodynamic parameters obtained during baseline: end-diastolic volume [Ved], end-systolic pressure [Pes] and volume [Ves], stroke volume [SV], stroke work [SW], maximum pressure rise per time unit [dP/dtMax] and slope of the preload recruitable stroke work [Mw]. Parameters for identifying the parabolic end-diastolic pressure-volume relationship (EDPVR [P0, λ, V0]), the arterioventricular coupling [Ees/Ea] and the pulmonary vascular bed [R, Zc, C] are presented.
